# Aberrant cytokine milieu and signaling affect immune cell phenotypes and functions in tuberculosis pathology: What can we learn from this phenomenon for application to inflammatory syndromes?

**DOI:** 10.1038/s41423-021-00695-8

**Published:** 2021-05-25

**Authors:** Ernest Adankwah, Julia Seyfarth, Richard Phillips, Marc Jacobsen

**Affiliations:** 1 Department of General Pediatrics, Medical Faculty, University Hospital Düsseldorf, Heinrich-Heine-University, Düsseldorf, Germany; 2 Department of Medical Diagnostics, College of Health Sciences, Kwame Nkrumah University of Science and Technology (KNUST), Kumasi, Ghana; 3 Department of Medicine, School of Medicine and Dentistry, College of Health Sciences, Kwame Nkrumah University of Science and Technology (KNUST), Kumasi, Ghana; 4 Kumasi Centre for Collaborative Research in Tropical Medicine (KCCR), Kumasi, Ghana

**Keywords:** Antimicrobial responses, Lymphocyte activation, Diagnostic markers

**Fig. 1:**
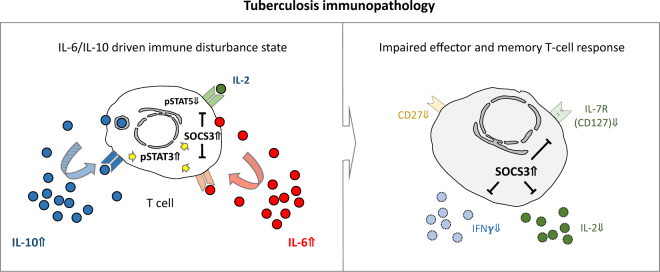
IL-6/IL-10-driven immune disorder state in acute tuberculosis and implications for effector and memory T-cell responses. This schematic depiction summarizes our hypotheses based on descriptive tuberculosis patient studies performed in Kumasi and the Ashanti region of Ghana. Potential triggers (i.e., IL-6 and IL-10) of immune disorder and direct effects on T cells (i.e., constitutively high pSTAT3 and SOCS3 expression) are shown on the left, and the immune phenotype and functional consequences are shown on the right. Impaired effector and memory T cell responses in tuberculosis include diminished IL-2 and IFNγ secretion by memory/effector T cells, reduced expression of the costimulatory receptor CD27 and decreased sensitivity to IL-7. Aberrantly high expression of SOCS3 was shown to be involved in the regulation of IL-7R (CD127) expression and was associated with low effector cytokine secretion of T cells from tuberculosis patients

Our previous study published in Cellular & Molecular Immunology (Harling et al.^1^) described aberrant immunological features in the pathogenesis of tuberculosis. Overall, we provided evidence supporting an impact chain regarding aberrant high interleukin (IL)-6 and IL-10 cytokine expression and respective receptor signaling affecting T-cell functions in acute tuberculosis. Constitutive STAT3 phosphorylation, high expression of its key regulator suppressor of cytokine signaling (SOCS)3 and spontaneous IL-6/IL-10 secretion characterized CD4^+^ T cells from tuberculosis patients. As a consequence, effector cytokine expression of *Mycobacterium (M.) tuberculosis*-specific memory T cells was potentially impaired by SOCS3 blocking IL-2-dependent STAT5 phosphorylation. Notably, based on principal component analysis, IL-6, IL-10, pSTAT3, and SOCS3 were the most influential factors that distinguished tuberculosis patients from healthy controls. The study by Harling et al. and others^2–5^ performed by our group in the same region in Ghana strengthened the assumption about generally impaired immune mechanisms in acute tuberculosis (depicted in Fig. 1). Phenotypic and functional changes in adaptive and innate immune cells provide a complex picture of tuberculosis pathognomonic mechanisms potentially caused by aberrantly high IL-6 and IL-10 levels. Here, we comment on key findings and the potential relevance for inflammatory syndromes in infectious diseases.

Tuberculosis is a chronic bacterial disease caused by mycobacteria from the *M. tuberculosis* complex. Transmitted via aerosol exhaled from patients with active pulmonary tuberculosis, *M. tuberculosis* infection remains asymptomatic in the vast majority of contacts. Immune surveillance of tuberculous granulomas is central to prevent dissemination and disease progression. Key immune factors (i.e., T cells, IFNγ, and TNFα) contribute to immune protection, and their deficiency or depletion due to interventions frequently leads to *M. tuberculosis* reactivation. However, there is increasing evidence that the immunopathology of tuberculosis is multifaceted and involves a broad spectrum of immune pathways. Heterogeneity of tuberculosis immunopathology is indicated by cohort differences in previous studies and limited common features identified in meta-analyses.^6^ Environmental factors (e.g., coinfections) and immune system genetics likely contribute to these differences. Against this background, we performed several studies, including the highlighted study by Harling et al., in the same region of central Ghana involving comparable cohorts of tuberculosis patients and asymptomatic controls.^1–5^

The study by Harling et al. was based on the hypothesis that pathognomonic features of tuberculosis are detectable in peripheral blood. Evidence supporting this assumption came from immune diagnostic tests (i.e., IFNγ release assays, IGRAs) for the detection of *M. tuberculosis* infection. IGRAs use short-term in vitro stimulation for the detection of recall T cell responses to *M. tuberculosis* antigens induced by previous *M. tuberculosis* infection. In addition to specific antigens (i.e., *M. tuberculosis* peptides/proteins), commercially available tests comprise ‘positive controls’ based on mitogen-induced T-cell cytokine production. Mitogen-induced T-cell responses are generally detectable, and the extent should not differ between matched study groups. However, we and others detected lower mitogen-induced IFNγ secretion in tuberculosis patients from Ghana than in healthy controls.^3,7^ Mitogen-induced IFNγ levels were below the detection limit for a subgroup of tuberculosis patients, which led to false-negative or ‘indeterminate’ IGRA results.^3^ These results suggested generally impaired T-cell cytokine expression as an immunopathognomonic feature in tuberculosis patients. This assumption was strengthened by T-cell phenotype analyses. T cells from tuberculosis patients showed aberrantly high expression of activation markers (i.e., CD38 and HLA-DR) and reduced levels of the TCR coreceptor CD27. Whereas this effect was initially hypothesized to be solely a feature of *M. tuberculosis*-specific T cells,^8^ we recently showed that the expression of CD27 was generally impaired in tuberculosis patients and that this effect was not restricted to *M. tuberculosis*-specific T cells.^2^ Similarly, we found lower IL-7 receptor (CD127) expression in T cells from tuberculosis patients than in T cells from controls.^5^ Aberrant phenotypes in tuberculosis pathology are not restricted to T cells but also affect innate immune cells such as monocytes. Monocyte subsets are characterized by the dominance of a CD14^+^/CD16^+^ phenotype, also termed ‘inflammatory monocytes’. We were able to confirm these findings recently and, moreover, detected reduced CD127 expression in monocytes from tuberculosis patients.^9^ As seen in T cells,^5^ CD127 signaling in monocytes was affected, and monocytes showed reduced ex vivo STAT5 phosphorylation.^9^ Decreased CD127 expression has functional implications for both T cells and monocytes. T cells were impaired for effector cytokine expression upon IL-7 costimulation during T-cell receptor activation,^5^ and monocytes had decreased antimycobacterial cytotoxicity in the absence of IL-7.^9^ Taken together, these studies suggested general immunopathognomonic features of tuberculosis and potential interrelationships that render single or few causative factors possible. To the best of our knowledge, the trigger(s) for aberrant immune cell phenotypes and associated functional differences in tuberculosis have not been identified.

Harling et al. provided initial evidence that serum cytokines, especially IL-6 and IL-10, are potential candidates.^1^ Aberrant serum cytokine levels have been described to play a role in tuberculosis pathology. IL-6 was previously identified as a pathognomonic marker, with high serum concentrations at acute tuberculosis stages and decreased levels during therapy and recovery.^10^ Recently, we showed that IL-6 is produced by T cells in response to acute *M. tuberculosis* antigens (i.e., ESAT6 and CFP10) in tuberculosis patients.^4^ Whereas healthy controls showed broader T-cell responses against *M. tuberculosis* latency-associated antigens, acute tuberculosis immune responses were dominated by T cell-produced IL-6.^4^ It is therefore tempting to speculate that IL-6-dominated T-cell responses against acute *M. tuberculosis* antigens are an initial trigger of immunopathology in tuberculosis patients.

The finding that the level of IL-10, a cytokine with immune regulatory functions, correlates with IL-6 expression and serum levels in tuberculosis is seemingly controversial. Concomitantly increased IL-6 and IL-10 serum concentrations in tuberculosis patients have been described,^10^ and Harling et al. demonstrated a correlation between IL-6 and IL-10 levels both spontaneously and after *M. tuberculosis*-specific stimulation.^1^ Similar to IL-6, IL-10 induces STAT3 phosphorylation and SOCS3 expression, but in contrast to IL-6, SOCS3 is not capable of inhibiting IL-10 receptor signaling. Therefore, persistent IL-10 signaling may contribute to constitutive STAT3 phosphorylation and aberrant SOCS3 expression.^1^ How constitutive pSTAT3 and high SOCS3 levels affect T cell function remains poorly defined. The study by Harling et al. revealed decreased *M. tuberculosis*-specific effector/memory T cell proportions with high SOCS3 expression. As potential mechanisms, interference of SOCS3 with IL-2-induced STAT5 phosphorylation was detected,^1^ and recently, we demonstrated that aberrant SOCS3 expression also affects CD127 reexpression during T cell activation.^11^ These findings strengthen the potential impact factor chain among IL-6/IL-10, pSTAT3/SOCS3 and impaired T-cell functions in tuberculosis (Fig. 1).

Evidence for a role of these mechanisms beyond tuberculosis comes from the field of inflammatory diseases, namely, rheumatoid arthritis (RA) and inflammatory syndromes. High IL-6 serum levels are also associated with constitutive STAT3 phosphorylation in patients with early RA. Strikingly, Anderson et al. detected a STAT3 target gene signature with aberrant expression, including that of SOCS3, PIM-1, and Bcl-3, in CD4^+^ T cells from RA patients.^12^ These molecules were also among the most highly expressed genes and part of a discriminatory gene signature in CD4^+^ T cells from tuberculosis patients.^13^

Inflammatory syndromes are receiving a great deal of attention, not least because of the SARS-CoV-2 pandemic. In tuberculosis, patients with HIV coinfection have a high risk of developing immune reconstitution inflammatory syndrome (TB-IRIS) during antiretroviral therapy. TB-IRIS is characterized by high IL-6 and IL-10 serum levels leading to disastrous inflammation.^14^ An aberrantly strong immune response against *M. tuberculosis* antigens is hypothesized to cause TB-IRIS. Although T cell-specific IL-6/IL-10 secretion has not been determined in TB-IRIS, triggers similar to those in acute tuberculosis patients may be assumed. In COVID-19, cytokine release syndrome (CRS) and multisystem inflammatory syndrome in children (MIS-C) occur during and after SARS-CoV-2 infections. Both CRS and MIS-C are characterized by high IL-6 and IL-10 serum concentrations, which serve as diagnostic criteria.^15^ In addition, constitutive pSTAT3 expression has been found in individual CRS patients.^15^ Aberrantly strong immune responses in patients with a high SARS-CoV-2 burden in combination with an initially inhibited IFNγ response were described as potential triggers of COVID-19-associated inflammatory syndromes.^16^ The clinical relevance of these findings has now been confirmed by treatment trials for CRS/MIS-C targeting the IL-6 receptor (tocilizumab).

Overall, there is increasing evidence of similarities between mechanisms causing tuberculosis immune pathology and inflammatory syndromes. Changes in serum cytokines, cytokine receptor signaling, immune cell phenotype, and subset distributions potentially contribute to and qualify as candidates for early prediction or biomarkers. These findings, centrally the one by Harling et al., can promote early diagnosis and target-directed immune modulatory therapies in the future.
